# Effect of Botulinum Toxin-A Injected to Muscle Tissue on Perfusion and Survival of Fasciocutaneous Single Perforator-pedicled Propeller Flap in Rats

**DOI:** 10.4274/balkanmedj.galenos.2019.2019.9.44

**Published:** 2020-02-28

**Authors:** Umut Zereyak, Neşe Kurt Özkaya, Zekiye Hasbek

**Affiliations:** 1 Clinic of Plastic Reconstructive Aesthetic Surgery, Sivas Numune Hospital, Sivas, Turkey; 2 Department of Plastic Reconstructive Aesthetic Surgery, Cumhuriyet University School of Medicine, Sivas, Turkey; 3 Department of Nuclear Medicine, Cumhuriyet University School of Medicine, Sivas, Turkey

**Keywords:** Botulinum toxin-A, flap necrosis, propeller-perforatorpedicled flap, rats, scintigraphy, ultrasonography

## Abstract

**Background:**

In plastic surgery practice, fasciocutaneous single-perforator-pedicled propeller flap is a preferred procedure; however, its survival rate is below than expected, especially in flaps with a big rotation arc. When botulinum toxin-A is injected into the muscle tissue that the perforator pedicle is arisen, the tonus of pertinent muscle can reduce and the blood flow of its perforator pedicle can increase. Therefore this procedure can improve the survival rate of single-perforator-pedicled propeller flap.

**Aims:**

To evaluate the effect of botulinum toxin-A injected with ultrasonographic guidance into the muscle tissue that the perforator pedicle is arisen from one month ago on the perfusion of flap scintigraphically and the survival rate of single-perforator-pedicled propeller flap in a rat model.

**Study Design:**

Animal experiment.

**Methods:**

Three study groups were receiving botulinum toxin-A (16 IU-0.4 mL), normal saline (0.4 mL), and no study drug one month ago before flap surgery. Injections were performed under ultrasonography guidance. Flaps were elevated fasciocutaneously over the right 2nd perforator pedicle, under the corneous, with a surgical loupe and microsurgery tool and were rotated clockwise 180°. Then the scintigraphic measurements were obtained after flap elevations in the study groups, including the whole-body and flap perfusions in the study rats. The involvement rate presents the ratio of flap perfusion to whole-body perfusion. Flaps were sutured back to the abdominal wall at the latest twisting angles. With standard photographs taken in all the groups on day 8 after the operation, whole and necrotic flap areas were calculated.

**Results:**

Scintigraphically the involvement rate (the ratio of flap perfusion to whole-body perfusion) of the flaps in the botulinum toxin-A group were found significantly higher than those in the other groups (p<0.05). The area of a flap in the botulinum toxin-A group on day 8 post flap suturing was found to be significantly higher than those in the other groups (p<0.05). The area of a necrosis and the percentage of necrosis on day 8 post flap suturing in the botulinum toxin-A group was found significantly lower than those of the sham and null groups (p<0.05).

**Conclusion:**

In a rat model, if with the ultrasonographic guidance, botulinum toxin-A is injected to the muscle which perforator of the prospective single-perforator-pedicled propeller flap originated and flap surgery is performed one month later after this injection, the perfusion of single-perforator-pedicled propeller flap increases scintigraphically and this improves flap survival and reduces its necrosis.

Fasciocutaneous single-perforator-pedicled propeller (SPPP) flaps are increasingly used in aesthetic and reconstructive surgery. Hyakusoku, in 1991, defined them as perforator vascular-based, 90-degrees rotated flaps (1). This technique was continuously improved in later years (2). Their advantage are: easy and fast detaching, circulation safe, a large rotation arc (3), the possibility of reconstructing the deficiency with needed tissue, limited donor-site morbidness, and repair the defect in only one session (4,5).

During the preparation of these flaps in clinical practice, their four sides are cut, and the flap is rotated around its perforator pedicle up to 180 degrees (6). Following rotation, a significant problem that is faced is the viability of lower flap, due to the single arteriovenous perfusion of the perforator flaps. Preparation of single-perforator pedicled propeller flaps requires the attentive dissection of their small perforator pedicles, and this can be performed with an unpredictable caliber. When flap preparation is unsuccessful, necrosis can be seen in the flap edges or in the entire flap. Many surgical techniques and pharmacological drugs or agents have been tried to reduce their failure rate and to improve their viability (7,8,9,10,11,12).

Botulinum toxin-A (BoTA) is one of the local pharmacological agents used to increase flap viability. In several studies, BoTA has often been applied to the flap or its subcutaneous tissue (11,12,13). BoTA increases flap viability by reducing the innervation of the related muscle and having a positive effect on the mobility of the muscle flaps (14). Progressively, perivascular application of BoTA increases the vessel diameter, tissue blood flow, and flap viability (12,13,15); this can be as a result of structural changes in vessels and activated angiogenesis as shown by histological studies (16,17,18). Flap survival, in these flap studies, was evaluated by various methods such as scintigraphy, photographic and histological analyses. In surgical practice, radiopharmaceuticals have been used in flap surgery to predict flap viability (19,20). The use of technetium-99m methoxyisobutylisonitrile (Tc-99m MIBI) scintigraphy is an important examination in the evaluation of cardiac perfusion, the survival of the flaps, and the many soft tissues such thyroid and parathyroid tumors (21,22,23).

BoTA-related literature supports that intramuscular injection of BoTA can reduce the contraction of muscle tissue and this can decrease its pressure on the perforator vessels. There is no clinical or experimental study investigating the survival of fasciocutaneous SPPP flap, after BoTA injection into the muscle from which perforator vessels arose. We think that the use of ultrasonography (US) may be advantageous to determine the perforator vessels of pedicle and to perform US-guided intramuscular injection of BoTA to obtain consistent results, and that the evaluation of blood flow by scintigraphy after flap elevation can provide important data to increase the prospect of flap survival. The purpose of this study was to determine whether US-guided BoTA injection, made to the muscle from which the perforator of the flap arose, can increase flap perfusion and improve its viability in a rat model of the fasciocutaneous SPPP flap.

## MATERIAL AND METHODS

### Animals

All experimental procedures used in this research were reviewed and approved by the Animals Research Ethics Committee of our university (12.04.2017-65202830 050.04.04-47). Twenty-four male Wistar albino rats, weighing 320 (±30) g, were used. Before the surgical intervention, rats were housed individually in a polycarbonate cage in a room, at 20-22 °C temperature, and under a 12 h light-dark cycle. The rats had free access to food and water.

### Experimental design

The rats were randomly allocated to 3 study groups with 8 animals in each group: BoTA, sham and null groups.

**Figure 1:**
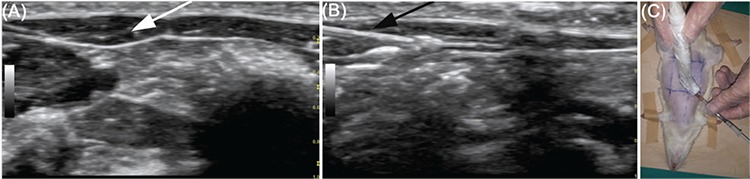
A representative ultrasonographic image of the rectus muscle from which perforator of the prospective SPPP flap originated (A). The white arrow shows muscle tissue. During BoTA injection, a representative ultrasonographic image of the needle and administered agent (B). A black arrow presents the needle. A representative rat photo during the ultrasonography of the flap side (C).

1. BoTA group: vials of lyophilized BoTA (Botox; Allergan, Irvine, CA, USA) was reconstituted in 2.5 mL of normal saline solution. A solution of 40 IU/mL of BoTA was obtained. In the BoTA group, under US guidance (Logiq E9, General Electric, USA), the muscle that second cranial perforator vessels of flap arose from was determined and 0.1 mL of BoTA (a total of 0.4 mL, 16 IU) was injected into the four quadrants of this muscle (Figure 1).

2. Sham group: The muscle from which the perforator of flaps arose was determined. Under US guidance (Logiq E9, General Electric, USA), the muscle from which second cranial perforator vessels of flap arose from was determined and 0.1 mL of normal saline (a total of 0.4 mL) was injected into the four quadrants of this muscle.

3. Null group: No injection was administered into the muscle tissue.

### Surgical procedure

In all the study groups, four weeks post the injection of the study drug, intraperitoneal ketamine (100 mg/kg) and intramuscular xylazine (25 mg/kg) were used for anesthesia before the surgical procedures and the rats were allowed to breathe spontaneously. They were stabilized on the operating tray in the supine position. The surgical sites were shaved, sterilized using povidone, and clothed by implementing principles of sterility. All surgical procedures were made by the same surgeon (U.Z.). A 4X surgical loupe (7033- OBO 4X Carl Zeiss, Germany) and standard microsurgery instruments were used.

**Figure 2:**
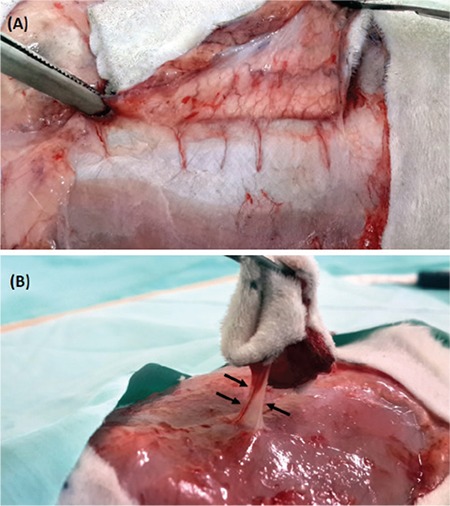
A representative image of an elevated flap with five perforator arteries (A). A representative image of the further prepared flap with a single perforator artery (black arrows) (B).

The flap boundaries were drawn with a ruler and pencil as the following: xiphoid lines as the upper limit and both the spinal iliac anterior superior as the lower limit and the axillary folds on the sides as the lateral limits. Following sterile isolation, the fasciocutaneous SPPP flap was designed on the anterior abdominal area. The flaps were prepared as second cranial pedicled perforator flaps based on a single perforator vessel from the rectus abdominis muscle, which was located just cranial to the major umbilicus and was standard in the rat. Flaps were elevated over the right 2^nd^ perforator pedicle, under the corneous, with a surgical loupe and microsurgery tool (Figure 2).

**Figure 3:**
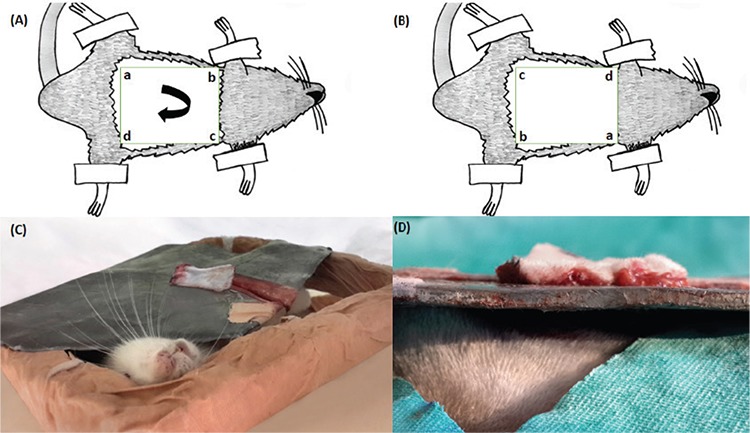
Drawings presenting 180° rotation of applied flap (A, B). Representative images of a rat in the apparatus designed to measure blood flow scintigraphically (C, D). This apparatus helps to protect the blood flow of flap from the pressure of the lead plate.

**Figure 4:**
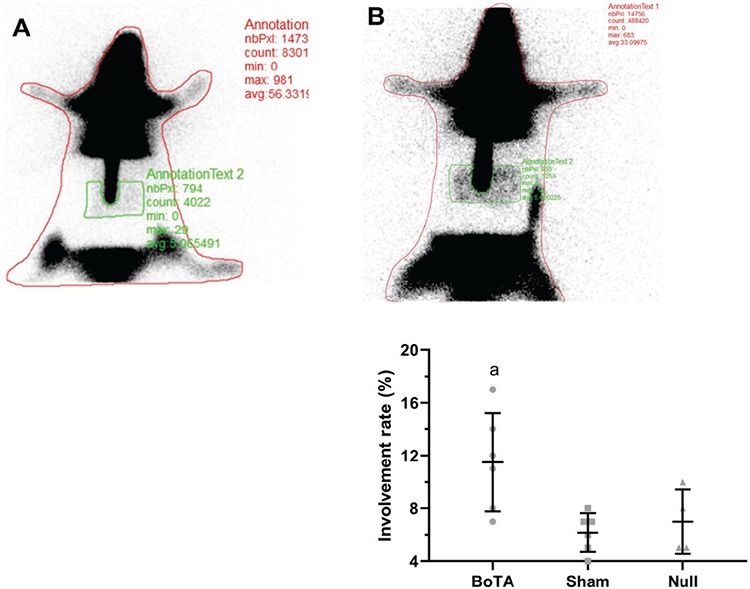
Representative scintigraphic images showing normal blood flow of flap in a rat receiving no BoTA injection (A) and presenting increased blood flow of flap in a rat receiving BoTA injection (B). The involvement rate of the BoTA group increased significantly compared to other groups (p<0.05). Whisker graph shows the scintigraphic measurements obtained after flap elevations in the study groups. The involvement rate presents the ratio of flap perfusion to whole-body perfusion. The involvement rate of the rats in the BoTA groups was found significantly higher than those of the sham and null rats (p<.05). Data were expressed as mean with SD. BoTA: botulinum toxin-A administered group, null: no injection group, SD: standard deviation, sham: normal saline-administered group

Then, the elevated flap was placed in the lead plate by rotating clockwise through 180 degrees. With the underlying foam apparatus, the pressure caused by the weight of the lead plate on the rats was prevented (Figure 3). 0.2 mL of mCi (Tc-99m MIBI) was injected into a tail vein for scintigraphic measurement of flap perfusion. Then, 5 minute images were taken using a low-energy high-resolution collimator in a double-headed gamma camera (DDD- Cor-Cam, Denmark). The acquisition time for the latter was 5 min, and the matrix was 256×256. The scintigraphy measurements were obtained after flap elevations in the study groups, including the whole-body and flap perfusions in the study rats. The involvement rate presents the ratio of flap perfusion to whole-body perfusion. The region of interest was drawn on the images of both the whole area of rats and flap area and Scintilation counts of all pixels were calculated for both whole area of rat and flap area with their values, the percentage of flap area was determined as the ratio of flap area in all rats (Figure 4). In two animals from the BoTA and sham groups, and three animals in the null group, scintigraphy data were not obtained because of technical problems; these animals were not excluded from the study to observe flap survival.

After scintigraphy, flaps were sutured back to the abdominal wall at the latest twisting angles with non-absorbable suture. For protecting the flap from external conditions, daily dressing was performed and to prevent suture lines from auto cannibalism, the body bandage was used. After surgery, each rat was placed in a separate cage with the same food supply and environmental fasciocutaneous condition. The rats were examined daily. There were no wound infections or complications observed as relevant to the outcome of the study.

### Image analysis

**Figure 5:**
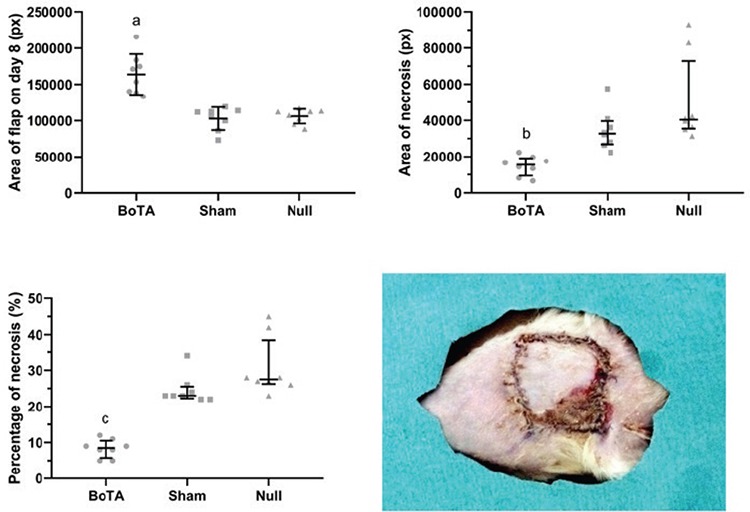
A representative image of normal and necrotic flap areas on day 8 later after flap suturing. Graphs present data of normal and necrotic flap areas and the percentage of necrosis seven days later after flap suturing. The normal flap area on day 8 later after flap suturing were expressed as mean with SD. One-way ANOVA test with post-hoc Tukey test revealed a significant difference among the study groups (p<0.05). The necrotic flap area and percentage of necrosis on day 8 later after flap suturing were expressed as median with interquartile range and Kruskal‒Wallis ANOVA test with post-hoc Dunn’s test revealed significant difference among the study groups regarding these variables (p<0.05). a, b, c p<0.05 vs sham and null. BoTA: botulinum toxin-A administered group, null: no injection group, SD: standard deviation, sham: normal saline-administered group

For the assessment of flap areas before surgery, images of surgical sites were obtained with a camera in macro shooting mode with 50 cm distance, 250 dpi resolution, and 6 mm focal length. Standard photographs were taken in all the groups on day 8 after the operation. For the assessment of normal and necrotic areas of flaps, the images of surgical sites were obtained with a camera according to the previously determined conditions, then normal and necrotic areas of flaps were analyzed with ImageJ software (imagej.nih.gov) and their areas calculated as pixel square (Figure 5). Then, the rats were sacrificed with intraperitoneally administered thiopental sodium solution (10 mg/kg).

### Statistical analysis

All statistical analyses were performed using IBM SPSS Statistical Software Version 22.0 (IBM, Armonk, NY, USA). The normality test was performed with the one-sample Kolmogorov‒Smirnov test. The area of the flap before elevation, necrotic flap area and percentage of necrosis on day 8 post flap suturing, was expressed as a median with interquartile range and analyzed with Kruskal‒Wallis ANOVA test, with post-hoc Dunn’s test. The normal flap area on day 8 later post flap suturing and involvement rate was expressed as a mean with standard deviation and analyzed with one-way ANOVA test, with post-hoc Tukey test. A p value less than 5% was accepted as statistically significant.

## RESULTS

**Figure 6:**
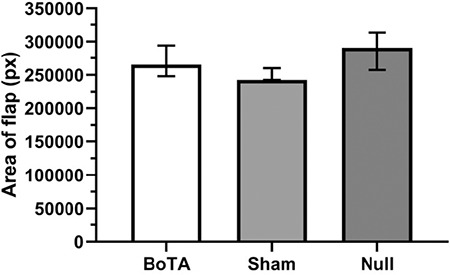
Area of flap bordered according to the determined landmarks before an elevation in the study groups. Areas were calculated as pixel values obtained from the picture of the surgical site. Data were expressed as median with interquartile range. Kruskal‒Wallis ANOVA test revealed no significant difference among the study groups (p>0.05). BoTA: botulinum toxin-A administered group, null: no injection group, sham: normal saline-administered group

There was no significant difference among the BoTA, sham and null groups with regard to the area of the flap before elevation [265284 (248007-294078), 242579 (242393-259922), 290649 (257459-313330), respectively; p>0.05]; and this may increase the reliability of the following flap related measurements (Figure 6).

The involvement rate of the flaps in the BoTA groups were found significantly higher than those of the sham and null groups (11.50±3.73 vs 6.17±1.47 and 7.00±2.45, respectively; p<0.05). There was no significant difference between the sham and null groups regarding the involvement rate (p>0.05) (Figure 4).

The area of a flap in the BoTA group on day 8 post flap suturing was found to be significantly higher than those of the sham and null groups (163726±27975 vs 102698±15670 and 105964±10027, respectively; p<0.05). The area of a necrosis on day 8 post flap suturing in the BoTA group was found significantly lower than those of the sham and null groups [15432 (9495-19159), 32718 (26812-3961), 40367 (35467-72795), respectively; p<0.05]. The percentage of necrosis in the BoTA group was found significantly lower than those of the other groups [8.50 (5.75-10.50), 23.00 (22.25-25.50), 27.50 (26.25-38.50), respectively; p<0.05]. There were no significant differences between the sham and null groups regarding the areas of flap and necrosis, and the percentage of necrosis (p>0.05) (Figure 5).

## DISCUSSION

In the current rat model, BoTA injection was made with the guidance of the US to the four quadrants of muscle, from which perforator of the flap arose and a month later, a fasciocutaneous SPPP flap (rotated 180° clock wisely) was prepared and its perfusion was measured by scintigraphy. Then, the flap was rotated 180° in the clockwise direction and sutured to its place, and 8 days later, whole and necrotic areas of the flap were measured with the help of photos obtained in a standard manner, for measurement with image analyses. Our findings revealed that BoTA injection made to the muscle, from which perforator of the flap arises, improved flap perfusion and survival. Measurements of flap areas before flap elevation were found to be comparable and this is a valuable finding that increases the reliability of other flap related measurements obtained after flap elevation.

The propeller flaps can be elevated easily and quickly during surgery, the circulation is safe and defect repair advantages can be counted as a single session (5). Propeller flaps are difficult to perform because of the anatomical variations in the tissues and the need for microsurgery when performing these flaps (6). In lower extremity reconstruction, it has advantages such as having higher rotation arch, lifting larger dimensions and lower donor area morbidity compared to other local flaps. This technique is a problem solver and is often preferred. In particular, the pedicle propeller flaps are used for reconstruction of defects in the distal lower leg and foot where the bone, tendon and nerves are exposed (24,25,26). This study may be a guide to reduce the complication rate, especially when reconstruction with propeller flap in the lower extremities where the pedicle originates between or into the strong and large muscle tissue.

Complication rates on the propeller flap have been obtained differently in many studies (24,25,26). The necrosis can only be limited by venous congestion or total flap necrosis can be seen. Multiple factors alone or in combination can influence the degree of necrosis: decrease of flap perfusion, vasospasm, flap contraction, thrombosis of flap pedicle, inadequate diameter of flap vessels for perfusion, decrease of perfusion due to twisting of flap vessels, extensive degree of rotation arc, and traction of flap pedicle. Especially, the reduction of blood volume of the flap is the main problem in propeller flaps and the most urgent solution is to reduce the tension that occurs when the twisting and flap are adapted to the new location. The rotation arch is important for perforator flap viability. This advantage ensures the propeller flaps to be applied differently in different areas. The complication rate for a 90 degree rotation in the SPPP flaps was found to be lower than the 91-180 degree rotation (24). The ability of a reliable 180-degree rotation is the matter that is widely discussed in the literature (25,26). The negative effect of twisting of pedicle on flap viability has been shown in some studies (27,28). Previous studies have evaluated the effect of pharmacological treatment for the prevention of flap necrosis caused by perforator pedicle twisting (8,9,11,13). Locally applied BoTA into flap is one of the molecules used to increase flap viability. The positive effect of preoperative subdermal BoTA applied to decrease necrosis has been showed (29). It has been shown that injecting BoTA, before the surgery, increases vessel diameter and blood flow velocity by chemical sympathectomy in the pedicle (15,29). In this study, BoTA was not applied to the flap or to subcutaneous tissue but the into rectus muscle, from which perforator of the SPPP flap arise. Flaps rotated by 180 degree were studied to shed light to the performance of flaps when their rotation degrees are high. For effective evaluation of the viable and necrotic areas, the flaps were designed as the largest sizes according to the size of the rats and the landmarks. The pedicle of the designed flap and the muscle, from which perforator of the flap arises, was determined by means of US and standardization, and a more controlled application was achieved.

BoTA injection showed that the muscle flap viability increases by reducing the innervation of the muscle, with BoTA having a positive effect on the mobility of muscle flaps (14). Progressively, perivascular application of BoTA increased the vessel diameter, tissue blood supply and subcutaneously increased flap viability compared to the control group (15). In the experimental study, increase in vessel diameters and angiogenesis was shown by histologic observation (16,17). Schweizer et al. (30) demonstrated that BoTA application to the vascular pedicle of an axial pattern flap leads to better flap perfusion, oxygenation, and flap viability. In the current study, the blood flow of flaps was evaluated by scintigraphy. The involvement rate of the flaps in the BoTA groups was found to be significantly higher than those of the other groups. In experimental studies, scintigraphy can be considered as a valuable method in the evaluation of flap blood flow. Also, the area of a flap after BoTA injection was considerably bigger and the percentage of necrosis was remarkably lower on day 8.

There are a few limitations of this study. While the elevated were rotated at 180 degrees to examine viability, the vascular pedicle was not in the center of flap. However, with the relaxant effect of BoTA on the muscle surrounding it, this did not lead to traction of the perforator vessels. Although there is a possibility of vasodilation of pedicle vessels after intramuscular BoTA injection, with US, we administered BoTA into the muscle tissue far from vascular pedicle. However, in the current study protocol, we thought there is no important contribution to the increased blood flow of perforator vessels, directly induced by BoTA injection. Further investigations may examine flap survival rates at higher rotational values. Histological evaluation and the amount of venous blood flow velocity can also be included in further studies. The effect of the optimal dose for using BoTA in flap surgery and its dose-response can be a subject of other studies. The radiopharmaceutical involvement was found at a higher rate in the flap model with a 180 degrees rotation. In this study, the US was used to choose the muscle of the perforator vessel. In future studies, the addition of blood flow measurement with pulsed Doppler US may provide a better result with the choice of the perforator vessel with more blood flow. Later on, areas that are difficult to repair can be included in clinical practice with BoTA applied perforator propeller flap.

In conclusion, with the current experimental settings, in a rat model, BoTA was injected, one month prior to the surgery, to the muscle which give rise to perforators. This resulted in increased flap perfusion and improved the survival of flap prepared as fasciocutaneous SPPP flap and rotated at 180° in the clock wise direction, before suturing its place on day 8. According to these findings, intramuscular BoTA injection, before the use of fasciocutaneous SPPP flap, has the potential to be used as an experimental drug in studies about flap survival and scintigraphy can be used as a modality to assess the perfusion of flaps before suturing its place.
